# Parisian Intelligence

**Published:** 1844-05-18

**Authors:** 


### PARISIAN INTELLIGENCE.

M. Guersant, senr., speaking of a patient then in one of his wards at the Children's Hospital, says, "if, in certain cases, typhus begins with diarrhœa, it is probably because, in addition to the inflammation of the extremity of the ileum and the other derangements, whether local or general, there exists inflammation of the mucous membrane of the large intestine; that the rumbling in the bowels is not peculiar to typhus, for it is observed in entero-colitis, and when the person has taken an enema a short time previous; that when typhus begins with dysenteria, nausea and vomiting do not exist; that sudamina are to be perceived not only in *this* disorder, but likewise in all those in which abundant perspirations take place; that the petechiæ, or papulæ, seldom appear before the second stage, and generally towards its close or at the beginning of the third ; that the dicrotic pulse is rarely to be met with in children, and exists principally in adults; that in fatal cases, patients are carried off as often by the derangement of the lungs as by the original malady ; and that the alteration in the blood is one of the causes which increases the danger attendant on bronchitis, by the congestion it favours, if not produces, of the lungs.

Professor Roux presented some reflections on the case of a patient operated upon some time ago for popliteal aneurism, by the ligature of the femoral artery, in consequence of several accidents which had developed themselves, and which rendered the ligature of the external iliac absolutely necessary. The wound was swoln, of a dark red colour, and bled copiously; above it was a considerable tumour, accompanied by a *pastiness* of the surrounding parts, and pulsations similar to those observed in aneurism, which ceased on pressure of the external iliac. The hæmorrhage proceeded, in all probability, from a small crack in the femoral artery, through which the blood escaped into the neighbouring cellular tissue, and formed a species of tumour. The swelling increasing in size, and the skin over it becoming erysipelatous, Professor Roux considered the ligature of the external iliac as the only means of averting the danger, "This," continued the skilful operator, "is the first time I have had an opportunity of performing it, notwithstanding my long surgical career. Abernethy was the first who, in the beginning of this century, performed the operation, but soon had numerous imitators in England and America, where popliteal aneurism, with a diseased state of the femoral artery, necessitating the ligature of the external iliac, is far more frequent than in France, probably on account of the greater muscular efforts to which workmen are exposed. As to the operation, the following ought to be preferred :—the parts are divided by a rectilinear incision, forming an acute angle with Poupart's ligament, taking care not to wound the epigastric or the circumflex iliac arteries, and their branches. The cylinder of Sparadrap is prejudicial by the phlegmonous inflammation to which it sometimes gives rise, and ought not, therefore, to be employed ; besides, the artery being constantly on the stretch, it will be advisable to place two ligatures on it as far apart as possible, and divide it in the interval, according to the method recommended by M. Maunoir, of Geneva. For some days the patient appeared to be improving; the temperature of the limb was natural, probably owing to the dilated state of the collateral branches produced by the first operation, when of a sudden he was siezed with violent hæmorrhage, To discover the cause, an incision was made over the femoral artery, by which a great quantity of blood escaped, and was stopped by the ligature of the vessels altogether. The patient, however, died 8 hours after, whether from the excessive loss of blood, or from the nervous disturbance caused by the operation, it is difficult to say. Consecutive hæmorrhage generally comes on at a much earlier period than in the present case, and is produced—1st, by accidents, or peculiar local causes ; 2d, by a predisposition, or species of general diathesis : in the former, it may be considered as primitive, in the latter, as secondary." *Post-morten* examination, performed by Professor Roux himself—dissection of the diseased limb : "The skin covering the popliteal aneurism was thinner than usual, and beneath it was a thin layer of the muscles of the thigh. The tumour about the size of the fist, when opened, was found to contain blood, having the consistence and appearance of currant jelly. It was situated at the beginning of the popliteal artery,—a circumstance seldom to be met with, the disease generally developing itself first lower down. The crural artery, dilated in the groin, was so firmly united to the vein and the surrounding parts, that it was difficult to say exactly whence the last hæmorrhage proceeded; the external iliac was in its normal state, and the two ends of the divided artery were easily discovered." In conclusion, the professor asked, "whether in cases in which the femoral artery is simply dilated, the ligature ought, in the first instance, to be placed on the external iliac? Without having any decided opinion on the subject, I should say, that it would be advantageous to begin by tying the femoral, in order to allow the collateral branches to become sufficiently large to carry on the circulation after the ligature of the external iliac, removing thus one of the causes of danger attendant upon the last operation. This idea deserves attention, the more so, as, at the present moment, the method preconised by Brasdor, and employed so successfully by Wardrop, seems to be coming into vogue."

Professor Velpeau in fractures of the neck of the femur, has, for the last 15 years, successfully adopted the mode of treatment recommended by Astley Cooper and Lallement, which consists in leaving the cure of the broken bone to nature: keeping the patient in bed for a fortnight, allowing him to sit up in an arm-chair, then to walk about with crutches, afterwards with a stick, and finally alone. The cure, by this method, is obtained, in the course of two months. In some cases, for instance in extra-capsular fractures, an apparatus may be employed, but splints ought to be rejected on account of the pressure they exercise on the parts. The dextrine bandage ought to be applied very carefully, so that, when dry, it may be sufficient to sustain the limb, and keep it in its natural position.

*Fractures of the Pelvis.*—One kind of fracture of this part, omitted by Boyer in his "*Traité des Maladies Chirurgicales*," is worthy of attention: viz. Fracture of the Acetabulum which Professor Velpeau has met with five or six times. It is generally caused, in young persons, by a fall on the knee aud feet, which, in an adult, would produce fracture of the neck of the femur.—*Symptoms*: Limb shorter than in its natuarl state, and cannot be extended; pain deeper seated than in fractures of the neck; foot turned inwards, but less than in the last disease; mobility of the pelvis. When free of complication, such as rupture of the bladder, rectum, uterus, or some other internal organ, or irritation produced by splinters of the bone, which are forced inwards, it may be considered as not very dangerous. In the treatment the only indication is to keep the pelvis perfectly motionless, by a bandage properly applied.

The same Professor has just published a memoir on the sub-cutaneous rupture or crushing of tumours in general, and principally of sanguineous tumours. The method consists in crushing the tumours by means of pressure exercised by the thumb, or, if that be insufficient, by placing on the swelling a flat piece of wood, on which a smart stroke may be given. Its great advantage is, that it cures the disease instantaneously, and ought therefore to be preferred to the simple or sub-cutaneous incision. The following conditions are necessary to render the cure more certain: the tumour ought to be placed on a surface which offers a certain degree of resistance—not to exceed the size of the fist—not to be of very long standing. Under similar circumstances, they are easier cured when accidental than when natural. By crushing an encysted tumour, it is brought to a state of infiltration, and is, therefore, easily absorbed. In concluding, the Professor passes in rapid review the different sorts of tumours to which this method may be applied, and says "that relapses generally take place in synovial and serous cysts,—that meliceris is refractory, as the contents cannot be absorbed,—that a steatoma my be cured by crushing, but only when small,—that when employed in enlarged lymphatic glands, the cure forms the exception.

---

Let the following be a warning to mothers, while suckling, not to give way to angry passions! A nurse, after going into a violent passion, on account of a family quarrel, gave the breast to her child, aged two months, and which had, until then, been in perfect health. Immediately after, the infant was seized with convulsions, which carried it off in the course of a short time.—*London Medical Times.*

